# Antifungal Activity of Essential Oils against* Candida albicans* Strains Isolated from Users of Dental Prostheses

**DOI:** 10.1155/2017/7158756

**Published:** 2017-09-26

**Authors:** Julliana Cariry Palhano Freire, José Klidenberg de Oliveira Júnior, Daniele de Figueredo Silva, Janiere Pereira de Sousa, Felipe Queiroga Sarmento Guerra, Edeltrudes de Oliveira Lima

**Affiliations:** ^1^Federal University of Paraíba, 58051-900 João Pessoa, PB, Brazil; ^2^Mycology Laboratory, Department of Pharmaceutical Sciences, Federal University of Paraíba, 58051-970 João Pessoa, PB, Brazil

## Abstract

**Objective:**

The objective of this study was to analyze the antifungal activity of citral, selected by screening natural products, against* Candida albicans* isolates from subjects who use dental prostheses.

**Methodology:**

Screening of essential oils, including those from* Mentha piperita* L. (Briq),* Origanum vulgare*, and* Zingiber officinale* L., and the phytoconstituents citral and limonene, to select an appropriate natural product. Citral, which mediated the best antifungal response, was selected for biological assays. The minimum inhibitory concentrations (MICs) and minimum fungicidal concentrations (MFCs) for citral and nystatin were determined by the microdilution method. Micromorphological analyses, time-kill curve, and modulation tests were performed.

**Results:**

The MIC and MFC of citral were established as 32 *μ*g/mL, consistent with fungicidal activity. The clinical strains were resistant to nystatin. Citral caused micromorphological alteration in the strains. In the time-kill curve, the growth of the clinical strain was reduction in growth equal to 3 log_10_ colony-forming units per milliliter after exposure to the MIC and MIC × 2 of citral for 2 h. Citral did not modulate the resistance of the studied strains to nystatin.

**Conclusion:**

This study revealed the potential of citral as a fungicidal agent and highlighted the resistance of clinical strains of* C. albicans* to nystatin.

## 1. Introduction

Denture stomatitis is a prevalent inflammatory reaction in patients using dental prostheses and* Candida* infection is its main etiological factor [1–3].* Candida albicans* is the most well-known fungal species associated with the clinical manifestation of this pathology. In addition to* C. albicans*, other less common* Candida *spp., such as* C. tropicalis*,* C. glabrata*,* C. parapsilosis*, and* C. krusei*, are pathogens present in the oral microbiota that can become pathogenic [4, 5].

Denture stomatitis associated with* Candida* spp. is difficult to treat and frequently presents with relapse [1].* Candida* infections have become a serious health problem, as fungi grow increasingly resistant to the available drugs, resulting in high relapse rates; new drugs must be researched and evaluated for their effectiveness in antifungal treatment [6–8].

In dentistry, herbal medicine has been used successfully for many years. Herbal medicines are a promising source of therapeutics in the pharmacological field [9–11]. Citral is a phytoconstituent present in several commonly used essential oils, such as the oil from* Cymbopogon citratus* [10]. Some pharmacological properties of citral have been reported in the literature, including antitumor [12], bronchodilatory [13], antiprotozoal [14], and antimicrobial [2, 3, 7, 15, 16] effects.

However, there are few studies on its mode of antifungal action and the effects of citral in combination with licensed drugs against clinical strains of* C. albicans* of prosthetic origin. In this context, the present study analyzed the antifungal activity of the phytoconstituent citral, selected from screening of 5 natural products, on strains of* C. albicans* isolated from subjects who use dental prostheses.

## 2. Materials and Methods

**Figure 1:**
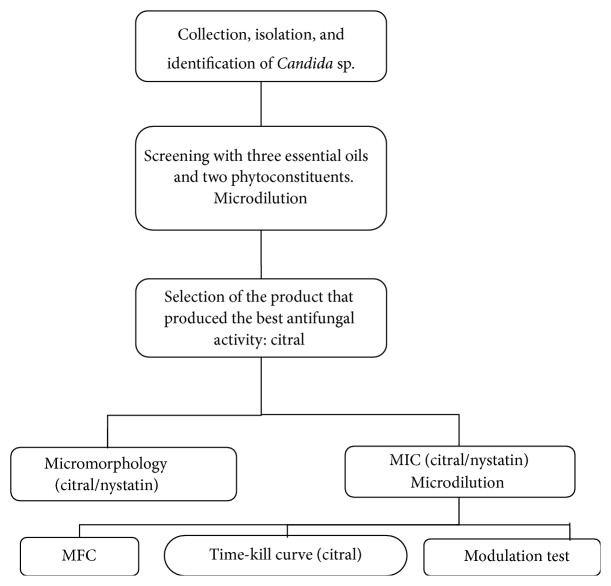
Flowchart of the experimental plane of the antifungal tests performed.

The present study was approved by the Research Ethics Committee of the Health Sciences Center, Federal University of Paraíba, under protocol number 0395/16, CAAE: 57435016.4.0000.5188. The biological material was collected in the Recanto do Poço community, Cabedelo, PB, Brazil. Laboratory tests were performed at the Mycology Laboratory, Federal University of Paraíba (UFPB). The methodological sequence used in the study is shown in Figure 1.

### 2.1. Collection, Isolation, and Identification of Biological Material

The study included 11 adult participants, 18 years of age and older, of both sexes, who used dental prostheses (total or partial, with or without a metal structure). The convenience sample consisted of 21 strains of* C. albicans*.

The strains of* C. albicans* were collected from the oral cavity and the prosthesis base of each research participant from August to November 2016. We isolated and identified the species according to the criteria established by Lodder [17], Hoog and Guarro [18], Kurtzmann and Fell [19], and Sidrim and Rocha [20].

We used 2 sterile swabs for each participant (Inlab Confiança, Brazil). The first, moistened in sterile physiological solution, was applied to the hard palate of the buccal cavity of the participant, swiped back and forth for 30 s, and then inserted in a test tube containing Sabouraud Dextrose Broth (SDA, Difco Laboratories, USA/France) for transport to the laboratory. The second swab was rubbed under the base of the prosthesis and immersed in a separate tube. The collected biological material was inoculated into 15 × 90 mm disposable Petri dishes (Inlab Confiança, Brazil) containing SDA (Difco Laboratories, USA/France), in the presence of 100 *μ*g/mL chloramphenicol (Sigma-Aldrich, St. Louis, MO, USA). After 24 to 48 h in a bacteriological oven at 35°C ± 2°C, we isolated the colonies with yeast-like fungi in CHROMagar™ Candida (Difco Laboratories, USA/France); after plaque growth, we evaluated the color and morphotypes of the colonies.

We identified the yeasts based on macromorphology, micromorphology, and physiological and biochemical tests. We analyzed germ tube production, yeast micromorphology on rice agar with TWEEN® 80, fermentation, and the assimilation of carbohydrates and performed molecular tests [20].

### 2.2. Fungal Strains and Inoculum Preparation

We used 20 clinical strains of* C. albicans* isolated from dental prosthesis users: LM-3B, LM-3P, LM-4B, LM-4P, LM-5B, LM-5P, LM-7B, LM-7P, LM-8B, LM-8P, LM-9B, LM-9P, LM-10B, LM-10P, LM-11B, LM-11P, LM-12B, LM-12P, LM-13B, and LM-13P. The species belonged to the Laboratory of Mycology, Department of Pharmaceutical Sciences, UFPB. The strains were maintained in SDA at 35°C ± 2°C.

Suspensions of the microorganisms were prepared in tubes containing 5 mL of sterile 0.9% saline solution (Farmax, Amaral, Divinopolis, MG, Brazil). The suspensions were shaken for 2 min with the aid of a vortex apparatus (Fanem, Guarulhos, SP, Brazil).

After shaking, the turbidity of each suspension was assessed and adjusted to that of a 0.5 McFarland barium sulfate suspension, which corresponds to an inoculum of approximately 10^6^ colony-forming units (CFU)/mL. This suspension was then diluted 1 : 10 with distilled water, resulting in an inoculum containing approximately 10^5^ CFU/mL, for use in the assays [21–24]. The final concentration obtained was approximately 1–5 × 10^5^ CFU/mL. Confirmation of the final concentration was achieved by counting the microorganisms in a Neubauer chamber.

### 2.3. Culture Media

RPMI 1640 (bicarbonate-free) (Sigma-Aldrich, Steinheim, Germany) and SDA (Difco Laboratories, USA/France) were used for the antifungal activity assays. The culture media were prepared according to the manufacturers' instructions.

### 2.4. Essential Oils, Antifungal Standards, and Preparation of Products

The natural products used in the screen and the biological assays are listed in Table 1. The product that produced the best antifungal response in the screen was selected for the other biological assays. Therefore, the test groups consisted of citral and the licensed drug nystatin (Sigma-Aldrich, São Paulo, SP, Brazil).

The natural products were solubilized in up to 10% dimethyl sulfoxide with 0.02% TWEEN 80 (Diadema, SP, Brazil). They were then emulsified with 3 mL sterile distilled water to obtain an initial concentration of 1,024 *μ*g/mL.

### 2.5. Screening and Determination of the Minimum Inhibitory Concentration

The screening of the natural products and determination of the minimum inhibitory concentration (MIC) of the selected product were performed using the microdilution technique, performed in triplicate, in sterile, U-bottom, 96-well microplates (Kasvi, Italy) [21, 23–26].

We added 100 *μ*L of 2× RPMI (Sigma-Aldrich, São Paulo, SP, Brazil) to each well of a plate. Subsequently, we dispensed 100 *μ*L of the 2× products into the wells of the first row of the plate; the products were serially diluted by the withdrawal of a 100 *μ*L aliquot from the concentrated well into a successor well, resulting in doubling dilutions from 1,024 *μ*g/mL to 128 *μ*g/mL for the screen and from 1,024 *μ*g/mL to 2 *μ*g/mL for the MIC determination. Finally, 10 *μ*L of the yeast inoculum was added to each well, such that each plate column contained a particular fungal strain.

Viability controls were performed on the fungal strains in the liquid medium under the same assay conditions. The plates were sealed and incubated at 35°C ± 2°C for 24 to 48 h. The MICs of the products used in the biological assays were defined as the lowest concentrations capable of visually inhibiting the fungal growth in the wells, as compared to the growth under control conditions.

### 2.6. Minimum Fungicidal Concentration

After determination of the MIC, 10 *μ*l aliquots of the supernatant from the wells corresponding to the MIC and the 2 immediately more concentrated concentrations (MIC, MIC × 2, and MIC × 4) were subcultured in a SDA containing plate, which was then incubated at 37°C for 24–48 h. The MFC was the lowest drug concentration that showed either no growth or fewer than three colonies. The assays were performed in triplicate, and the geometric mean values were calculated [27]. The MFC/MIC ratio was calculated to determine if the substance had fungistatic (MFC/MIC ≥ 4) or fungicidal (MFC/MIC < 4) activity [28].

### 2.7. Micromorphology Analysis

To study possible alterations in the micromorphology of* C. albicans* cells exposed to citral, we microcultured the samples on a slide in a Petri dish (camera wet) [29]. The molar agar-fubah-TWEEN 80 culture medium was fractionated into sterile tubes containing the MICs of the test products. A tube with the culture medium alone was used as the control. After homogenization, each culture medium was spread on a glass slide.

*C. albicans* in SDA were seeded on the slides and incubated at 35°C ± 2°C for 24 to 48 h. The slides were analyzed by light microscopy, at a magnification of 400×, to observe the formation of characteristic structures, such as blastoconidia, pseudohyphae, and chlamydoconidia.

### 2.8. Time-Kill Curve

We assessed the effects of the test product on the time-kill curves of the fungal strains using the methodology described by Klepser et al. [30], with some improvements.

For the analysis of microbial death kinetics, we selected 2 strains: an American Type Culture Collection standard (ATCC 76645) and a clinical strain, LM-8B, which demonstrated sensitivity to citral. In this assay, we observed the behavior of the selected yeast strains over 24 h in the presence of the MICs of citral.

Initially, 100 *μ*L RPMI 1640 (Sigma-Aldrich, São Paulo, SP, Brazil) was added to each well of a 96-well, U-bottom microplate with 10 *μ*L of the supernatants with various citral concentrations (MIC, MIC × 2, and MIC × 4) and incubated for 24 to 48 h at 35°C ± 2°C.

The inoculum was plated on a Petri dish (Alamar, Diadema, SP, Brazil) containing SDA (Difco Laboratories, Detroit, MI, USA) culture medium. A 10 *μ*L aliquot of the inoculum was removed from the microplate with a calibrated bacteriological loop (Inlab Confiança, Brazil) and then uniformly streaked along the surface of the SDA culture medium in the Petri dish at 0, 2, 4, 8, 12, and 24 h. The inoculated dishes were incubated at 35°C ± 2°C for 48 h.

The experiment was carried out in triplicate. The curves were plotted as the colony count (log_10_ CFU/mL) as a function of time (h) with GraphPad Prism 5.0 (GraphPad for Windows, San Diego, CA, USA). Fungicidal activity was classified as a reduction in growth ≥3 log_10_ (≥99.9%) and fungistatic activity as a reduction in growth of <3 log_10_ (<99.9%) CFU/mL, compared with the initial inoculum [30].

### 2.9. Modulation Test

To evaluate the modulatory action of citral on the resistance of* C. albicans* strains to nystatin, we determined the MIC of nystatin, using the microdilution technique in a sterile, 96-well, U-bottom plate (Kasvi, Italy), in the presence of a sub-MIC of citral (MIC/8) [31, 32].

The citral was emulsified at 4 *μ*g/mL in RPMI 1640 medium for a final 100 *μ*g/mL solution. This solution was added to each well of the microplate. Subsequently, 100 *μ*g/mL of nystatin was dispensed into the wells of the first row followed by microdilution, in order to obtain concentrations of 1,024 *μ*g/mL to 4 *μ*g/mL. Finally, 10 *μ*L of the yeast inoculum was added to the wells, such that each column contained 1 particular strain.

The MIC values of the standard antifungal were then compared in the absence and presence of the MIC/8 of citral. The plates were incubated for 24 to 48 h at 35°C ± 2°C for the evaluation of fungal growth. The tests were performed in triplicate.

## 3. Results and Discussion

Citral mediated the best antifungal response against* C. albicans* of the 5 screened products, which had been selected based on the ethnobotanical literature [33–35]; it inhibited the fungal strains at a concentration of 128 *μ*g/mL (Table 1).

All of the yeast strains were resistant to ginger essential oil. Sharifzadeh and Shokri [36] found that the MIC of ginger essential oil was 2,500 *μ*g/mL for* C. albicans* strains that were resistant or sensitive to fluconazole. Aghazadeh et al. [37] determined the MIC of ginger extracts as 5 mg/mL. We found that oregano oil had a MIC of 256 *μ*g/mL, in agreement with the study by Sharifzadeh and Shokri [36], which determined the MIC as 300 *μ*g/mL. Mint essential oil and the phytoconstituent limonene both had MICs of 256 *μ*g/mL.

As shown in Table 2, with the exception of its MIC against* C. albicans* LM-3B, the antifungal activity of citral was determined as 32 *μ*g/mL (MIC 90%). This MIC was similar to that (64 *μ*g/mL) determined by Leite et al. [15] against strains of* C. albicans* isolated from the blood. Lima et al. [7] found that 512 *μ*g/mL citral inhibited all* C. albicans* yeasts isolated from the blood, urine, respiratory tract, and vaginal secretions, suggesting higher resistance in strains isolated from those sites rather than from the oral cavity.

The citral MFC coincided with its MIC in the samples tested. According to Siddiqui et al. [28], a substance has fungistatic activity when the MFC/MIC ratio is ≥4 and fungicidal activity when the MFC/MIC ratio is <4. Hafidh et al. [38] characterized an MFC/MIC ratio between 1 : 1 and 2 : 1 as fungicidal activity, whereas they ascribed fungistatic activity to a ratio greater than 2 : 1. According to these methodologies, we observed fungicidal activity by citral. This finding corroborates the findings of Leite et al. [15] that the MFC of citral corresponded to its MIC (64 *μ*g/mL) for all the strains analyzed. Sousa et al. [16] observed MFC values between 256 *μ*g/mL and 1,024 *μ*g/mL (MFC_50_ and MFC_90_, resp.) for citral against* C. tropicalis* isolated from the blood, indicative of fungicidal activity against the tested yeasts.

**Figure 2:**
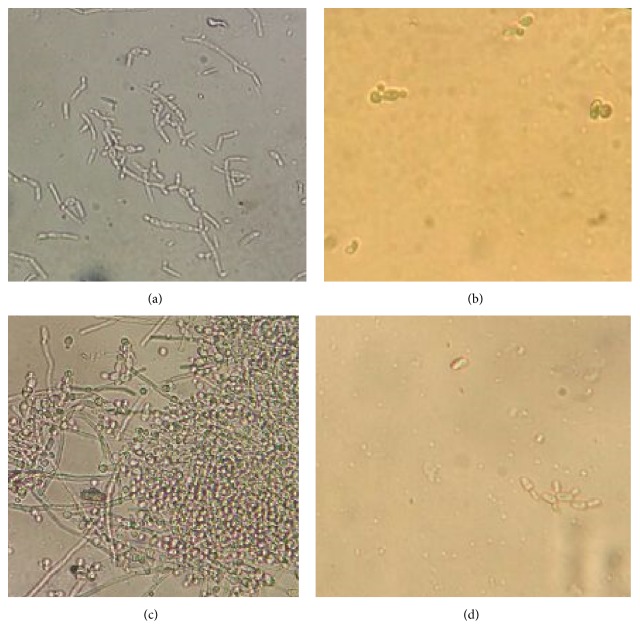
Micromorphology of* C. albicans* in absence (control) and presence of citral. (a)* C. albicans* ATCC 76645 in the absence of the product, demonstrating structures such as blastoconidia and pseudohyphae. (b)* C. albicans* ATCC 76645 in the presence of the phytoconstituent, rare yeasts, and pseudohyphae are observed. (c)* C. albicans* LM-11P in the absence of citral exhibiting large amounts of cell structures, blastoconidia, and pseudohyphae. (d)* C. albicans* LM-11P under the action of citrine MIC, with rare yeasts and pseudohyphae.

The micromorphological evaluations (Figure 2) of* C. albicans* ATCC 76645 and LM-11P under an optical microscope revealed fungal growth, characterized by the formation of pseudohyphae and blastoconidia. In the presence of citral, we observed few growth-related structures, with more blastoconidia than pseudohyphae, indicating that citral altered the morphology of the strains. According to Alves et al. [39], specific morphological changes are associated with the pathogenicity of microorganisms, as local environmental factors can alter the status of commensal fungi, thereby making them infectious. The yeast-hyphal morphological transition is relevant for the virulence of fungal infections. Leite et al. [15] also observed that* C. albicans* strains had few blastoconidia and lacked pseudohyphae in the presence of citral. Thus, these results are relevant to the development of a novel antifungal agent.

**Figure 3:**
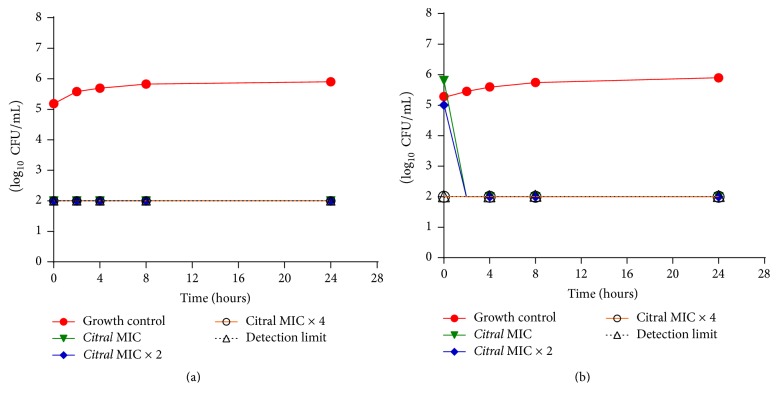
(a) Time-death curve for* C. albicans* ATCC 76645 when exposed to various concentrations of citral. (b) Time-death curve for* C. albicans* LM-8B under different concentrations of citral.

We analyzed yeast growth as a function of time in the presence of various citral concentrations. Two strains of* C. albicans* were subjected to microbial death kinetics analysis (Figure 3). We assessed the viable cell count over time to verify the fungicidal or fungistatic activity of the test product and to evaluate the interaction of the test product with the microorganism, in order to characterize the dynamic relationship between the product concentration and length of exposure [40]. We found a reduction in the growth of the clinical strain equal to 3 log_10_ CFU/mL after 2 h of exposure to the MIC and MIC × 2 of citral, classified as a fungicidal effect according to the criteria of Klepser et al. [40]. We did not observe growth of the clinical strain at any of the time points after exposure to the MIC × 4 of citral, or growth of the standard strain at any of the citral concentrations. These results revealed the concentration-dependence of the fungicidal activity of citral.

Leite et al. [15] and Zore et al. [3] also observed the fungicidal effect of citral on* C. albicans* using microbial death kinetics analysis. In the first study [15], the time required for citral fungicidal activity was 4 h at the MIC (64 *μ*g/mL) and 2 h at the MIC × 2. In the latter study, 640 *μ*g/mL citral killed 99.9% of the inoculum in 2 h.

The possible mechanisms of action of citral in fungal cells have not yet been fully elucidated. Park et al. [41] observed that citral was able to cause hyphal degeneration, cell wall membrane separation, and mitochondrial disintegration in the fungal species* Trichophyton mentagrophytes*. According to Harris [42], citral acts predominantly on the fungal cell membrane, affecting its structure and causing cell death by inhibiting spore germination, proliferation, and cellular respiration.

Lima et al. [7] and Leite et al. [15] concluded that the MIC of citral was not altered by the presence of sorbitol, suggesting that it does not act by modifying the fungal cell wall or by binding to ergosterol, but likely affects another target.

Most of the strains were resistant to nystatin, so it was not possible to determine a MIC; the exception,* C. albicans* ATCC 76645, was sensitive to a concentration of 256 *μ*g/mL citral.

The resistance of* Candida* spp. to nystatin has been documented in the literature; Sousa et al. [16] observed growth of all* C. tropicalis* yeasts isolated from the blood in the presence of fluconazole, except for* C. tropicalis* ATCC 13803. The authors emphasized that this drug is widely used in clinical practice because of its efficacy and low toxicity. Due to frequent exposure to antifungal drugs,* Candida* isolates and their species are regularly developing resistance.

The relationship between exposure and resistance may also explain the results of this study; the frequent use of nystatin or other drugs prescribed as antifungals for denture stomatitis associated with* Candida* spp. has made* C. albicans* strains resistant to nystatin [6–8]. Microbial interactions in the oral cavity favor the proliferation of microorganisms, which facilitates the development of* Candida* drug resistance [43, 44].

The resistance of clinical strains of* C. albicans* isolated from prosthesis users to the antifungal standard nystatin, which is used for the treatment of* Candida*-associated denture stomatitis, demonstrates the importance of investigating the efficacy and mode of action of novel products on this clinically relevant pathogen. Such research will facilitate the discovery of new antifungal agents.

The standard yeast ATCC 76645 possesses fewer fungal structures, like pseudohyphae and blastoconidia, than strains of clinical origin (Figures 2(a)–2(c)). This strain has not interacted with other microorganisms, unlike clinical* C. albicans *isolates. This characteristic may account for the sensitivity of* C. albicans* ATCC 76645 to nystatin; by contrast, the clinical strains were unaffected by this antifungal, resulting in growth at the concentrations studied.

The addition of citral to the growth medium at a subinhibitory concentration of 4 *μ*g/mL (MIC/8) did not alter the resistance of the clinical strains or the standard to nystatin, as assessed by proliferation. Furthermore, there are no studies in the literature investigating the ability of citral to modulate nystatin activity. The ability of unconventional compounds to increase the antimicrobial activity or reverse the resistance of microorganisms to drugs classifies the compounds as modifiers of antifungal activity [31]. Citral was not classified as a modifier of resistance to the antifungal nystatin in the studied strains.

This laboratory study has limitations. Given the increase in fungal infections and the high prevalence of denture stomatitis associated with* Candida*, the search for new, alternative treatments for these pathologies is necessary. Citral may be a promising candidate, as it mediated antifungal activity against clinical strains of* C. albicans* isolated from dental prosthesis users.

## 4. Conclusions

Among the natural products analyzed, citral produced the best antifungal response. Citral demonstrated fungicidal activity against* C. albicans* yeasts isolated from dental prosthesis users, as well as against the standard strain. The phytoconstituent caused morphological modifications to the yeasts studied.

Citral was distinguished by its promotion of microbial death in clinical* C. albicans* within 2 h of exposure, at both the MIC and MIC × 2. We verified the resistance of all of the clinical strains to nystatin. Citral did not alter the resistance of the strains to the licensed drug.

Citral is a promising phytoconstituent; future research should assess its mode of action against fungi and its cytotoxic potential, to facilitate the development of new antifungal agents against* C. albicans* spp.

## Figures and Tables

**Table 1:** Screening results for evaluation of the antifungal response of the products. Microdilution technique (1024 *μ*g/ml to 128 *μ*g/ml).

Essential oil					
Scientific name	Family	Popular name	Batch	Fabricator	MIC (*µ*g/mL)
*Origanum vulgare *L.	Lamiaceae	Oregano	0149/05209/F	Sigma-Aldrich, São Paulo, SP, Brazil	256
*Zingiber officinale*	Zingiberaceae	Ginger	0717/05209/F	Quinari Fragrâncias e Cosméticos Ltda., Ponta Grossa, PR, Brazil	+
*Mentha piperita *L.	Lamiaceae	Peppermint	0809/05209/F	Sigma-Aldrich, São Paulo, SP, Brazil	256

Phytoconstituent					
Name	Molecular formula		Batch	Fabricator	MIC (*µ*g/mL)

Citral	C_10_H_16_O		STBC5273V	Sigma-Aldrich, São Paulo, SP, Brazil	128
Limonene	C_10_H_16_		58296DK	Sigma-Aldrich, São Paulo, SP, Brazil	256

+: growth of microorganism.

**Table 2:** Results of MIC and MFC evaluation of citral and nystatin and MIC/MFC ratio of citral on *C. albicans*.

*C. albicans*	Citral (*μ*g/mL)				Nystatin (*μ*g/mL)	Control of strains	Control of the culture medium
	MIC	MFC	MFC/MIC	Activity antifungal	MIC		
3B	64	64	1	Fungicide	>1024	+	−
3P	32	32	1	Fungicide	>1024	+	−
4B	32	32	1	Fungicide	>1024	+	−
4P	32	32	1	Fungicide	>1024	+	−
5B	32	32	1	Fungicide	>1024	+	−
5P	32	32	1	Fungicide	>1024	+	−
7B	32	32	1	Fungicide	>1024	+	−
7P	32	32	1	Fungicide	>1024	+	−
8B	32	32	1	Fungicide	>1024	+	−
8P	32	32	1	Fungicide	>1024	+	−
9B	32	32	1	Fungicide	>1024	+	−
9P	32	32	1	Fungicide	>1024	+	−
10B	32	32	1	Fungicide	>1024	+	−
10P	32	32	1	Fungicide	>1024	+	−
11B	32	32	1	Fungicide	>1024	+	−
11P	32	32	1	Fungicide	>1024	+	−
12B	32	32	1	Fungicide	>1024	+	−
12P	32	32	1	Fungicide	>1024	+	−
13B	32	32	1	Fungicide	>1024	+	−
13P	32	32	1	Fungicide	>1024	+	−
ATCC 76645	32	32	1	Fungicide	256	+	−

+: growth of microorganism; −: without microorganism growth.
